# Antimicrobial Prescribing Practices Among Sri Lankan Veterinarians for Common Medical Conditions in Companion Animals

**DOI:** 10.3390/ani15010069

**Published:** 2024-12-31

**Authors:** Lalanthi Premaseela De Silva, Nayana Wijayawardhane, Ruwani S. Kalupahana, Kottawattage S. A. Kottawatta, P.G. Anil Pushpakumara, Christopher B. Riley

**Affiliations:** 1 Faculty of Veterinary Medicine and Animal Science, University of Peradeniya, Peradeniya 20400, Sri Lanka; lalanthidesilva@vet.pdn.ac.lk (L.P.D.S.); nayanaw@vet.pdn.ac.lk (N.W.); rskalupahana@vet.pdn.ac.lk (R.S.K.); sanda@vet.pdn.ac.lk (K.S.A.K.);; 2 Department of Clinical Studies, Ontario Veterinary College, University of Guelph, Guelph, ON N1G2W1, Canada; 3 School of Veterinary Science, College of Science, Massey University, Palmerston North 4410, New Zealand

**Keywords:** veterinarian, companion animal, dog, cat, antimicrobial, resistance, stewardship, prescription

## Abstract

Veterinarians are essential in antimicrobial stewardship. Companion animal (CA) practitioners have recently received more attention. There are few relevant studies on CA antimicrobial prescribing practices in South Asia. A cross-sectional survey on the antimicrobial prescribing practices of Sri Lankan veterinarians associated with six common CA infections and factors associated with bacterial culture and antimicrobial susceptibility testing (AST) was conducted. A survey of antimicrobial prescribing practices was distributed via email or professional events among veterinarians treating CAs. There were 127 respondents. Most were from CA-only practices (63%); the remainder worked in government, mixed, or other practices. Based on the World Health Organization’s criteria, 1.7% (13/783) of cases were treated with drugs authorized for human use only; 23.9% (187/783) were treated with highest-priority critically important antimicrobials; 4.7% (37/783) were treated with critically important antimicrobials, and 66.8% (523/783) were treated with highly important antimicrobials. The antimicrobial treatment of abscesses, skin, urinary, and ear infections was usually empirical, with only 12.4% (72/579) of cases utilizing AST to guide diagnosis and treatment. The use of AST was significantly associated with the type of antibiotic prescribed, the medical condition, the practice type, and continuing professional development, with the latter recognized as a pathway toward improved veterinary antimicrobial stewardship and the implementation of a One Health approach.

## Simple Summary

The appropriate use of antibiotics among companion animal (CA) practitioners has recently been a subject of interest. Surveys can assist in understanding the role of CA veterinarians in antimicrobial use. This study aimed to survey the antimicrobial prescribing practices of Sri Lankan veterinarians associated with common dog and cat bacterial infections of the skin, ear, and urinary tract, and to identify factors associated with bacterial culture and antimicrobial susceptibility testing (AST) to aid in the selection of the most appropriate antibiotic treatment. A survey was distributed among Sri Lankan veterinarians. Most veterinarians were from CA-only practices (63%); the remainder worked in government, mixed, or other practices. Based on the World Health Organization’s criteria, 2% (13/783) of cases were treated with drugs authorized for human use only; 24% (187/783) were treated with highest-priority critically important antimicrobials; 5% (37/783) were treated with critically important antimicrobials, and 67% (523/783) were treated with highly important antimicrobials. The antimicrobial treatment of abscesses, skin, and urinary and ear infections was based on AST for only 12% (72/579) of animals. When used, AST was significantly associated with the prescribing of tetracyclines, ear or urinary tract infections, the practice type, and continuing professional development. Education is recognized as a pathway toward improved veterinary antimicrobial stewardship and part of One Health programs.

## 1. Introduction

In 2019, there were 2300 human deaths attributable to, and 8800 associated with, antimicrobial resistance (AMR) in Sri Lanka [1]. This is within the 4.95 million who died globally from drug-resistant infections, including 1.27 million directly from AMR, in 2019 [1]. The emerging threat to Sri Lanka posed by AMR led to the development of the first National Strategic Plan for Combating Antimicrobial Resistance in Sri Lanka 2017–2022, which included enabling the surveillance of AMR in terrestrial and aquatic animals, an analysis of antimicrobial use in these species, and regulating and controlling the manufacture, import, export, and sale of veterinary drugs [2]. The latter measures were focused on food animals; this strategy did not target companion animals and their veterinarians. However, there is increasing concern that AMR organisms and their genetic elements may be transferred from dogs and cats to humans [3,4,5], contributing to increased human exposure to resistant pathogens. Recognizing the vital role of companion animals as part of the One Health paradigm, the 2023 update to Sri Lanka’s national strategic plan now specifically addresses infection prevention, antimicrobial use guidelines and regulations, and antimicrobial stewardship in these species [6].

In Sri Lanka and other Southeast Asian countries, the practical application of interventions to minimize the impact of AMR has been hindered by limited technical capacity, economic constraints, regulatory frameworks, and slow behavioral changes across all levels of antimicrobial stewardship, as well as the COVID-19 pandemic [7]. Veterinarians play an essential role in antimicrobial stewardship that has long been recognized in the agricultural sector, but only recently has attention been paid to companion animal practice [8]. To understand the role of veterinarians in antimicrobial stewardship and their prescribing practices, there have been many international surveys of companion animal veterinarians [8,9,10,11,12,13,14,15,16,17], but few are relevant to South Asia [18,19]. In the United Kingdom, veterinary prescribing practices, interactions with clients, infection control practices, and the use of diagnostic tests to confirm infection have been identified as influential factors in reducing AMR and gains in antimicrobial stewardship [20]. This echoes concerns that were identified in a qualitative study on 29 Sri Lankan healthcare professionals, including 10 veterinarians, and their antimicrobial stewardship practices [21]. These authors identified inappropriate antimicrobial prescribing practices, a variable understanding of the factors leading to AMR, challenges in resisting client expectations and demands for antibiotics, the influence of national and multinational pharmaceutical companies, and lack of regulation or enforcement to prevent the illegal or inappropriate prescription and distribution of these drugs. Therefore, given these barriers, a deeper understanding of prescribing practices among Sri Lankan veterinarians who treat companion animals and the factors that influence inappropriate antimicrobial use is needed. A contemporary web-based survey of young Sri Lankan veterinarians identified low rates of sample submission for antimicrobial susceptibility testing and the frequent inappropriate use of antimicrobials [19]. The quantitative analyses of associated factors in this study were limited, and the multivariable modeling of associated factors was not performed.

The objective of the current study was to conduct a cross-sectional study of the antimicrobial prescribing practices of Sri Lankan veterinarians associated with treating pyodermas, skin wounds, abscesses, urinary tract infections, and ear infections in companion animals. A second objective was to identify factors associated with the submission of samples by veterinarians for bacterial culture and susceptibility testing. The authors’ overarching goal was to obtain data that would contribute to developing antimicrobial stewardship guidelines for veterinarians treating companion animals in Sri Lanka.

## 2. Materials and Methods

### 2.1. Target Population

The target population comprised Sri Lankan veterinarians registered with the Veterinary Council of Sri Lanka involved in companion animal practice, solely or as a part of their practice. Government veterinarians involved in administrative authorities and exclusive farm animal practice were excluded from the study population. Based on the information from Department of Animal Production and Health, Sri Lanka, the estimated number of veterinarians involved in companion animal practice in Sri Lanka at the time of the survey was 890 of 2041 gazetted veterinarians. A representative sample size was estimated at 268 respondents to attain a 95% confidence level with a sampling error of 5% using an online random survey calculator [22].

### 2.2. Survey Design and Distribution

The cross-sectional survey comprised questions addressing the veterinarian’s educational background, geographic location, participation in continuing professional development, the use of culture and susceptibility testing and antimicrobial prescribing patterns for commonly encountered bacterial infections in companion animals. The questionnaire was distributed to a convenience sample of veterinarians who were invited to voluntarily participate in the anonymous survey via email or in person at workshops and CPD programs conducted by the Society of Companion Animal Practitioners (SCAP), Sri Lanka. Following informed consent, veterinarians were offered the opportunity to self-administer the survey. Respondents who volunteered to participate were asked to describe antimicrobial choices and duration of therapy for their most recent cases of acute pyoderma, recurrent or deep pyoderma, a skin wound, a cat bite abscess, a urinary tract infection, and an ear infection. Respondents were asked whether bacterial cultures and susceptibility testing were performed and about the duration and type of antimicrobial therapy used. After giving informed consent, veterinarians were offered the opportunity to self-administer the survey. The survey was distributed between January 2020 and June 2021. The questionnaire is available in the Supplementary Materials, Table S1.

### 2.3. Data Analysis

Surveys in which the respondent had completed one or more sections of the questionnaire were retained for analysis. Survey data were transcribed and entered into a spreadsheet (Microsoft Excel, Version 2312, Microsoft Corporation, Mississauga, ON, Canada). Data were checked for errors. Respondents were asked to classify their field of practice as companion animal (CA), mixed practice (MP), government practice (GV), or other. Those that indicated multiple practice fields CA-GV (n = 1), CA-MP (n = 1), and GV-MP (n = 1) were reclassified to CA, MP, and GV, respectively, before further data analysis was performed. For variables where a numerical response was requested, but the respondent gave a value range, the mid-value of the range was used for further analysis.

#### 2.3.1. Duration of Antimicrobial Treatment

For questions about the antimicrobial treatment of the most recent case with a specific diagnosis, 58/127 listed 2 to 7 different antibiotic treatments for the same case. Only the first antimicrobial treatment was used for the following analysis, which included only respondents who described the treatment of two or more of the conditions queried. The numerical data for the duration of antimicrobial therapy were not normally distributed, so Kruskal–Wallis rank sum tests were performed to determine whether there were significant differences (*p*-value < 0.05) in the duration of antimicrobial treatment associated with the different conditions for which therapy was recommended, the predominant field of practice of the respondent, and whether or not the respondent had postgraduate training (PGT) or had completed continuing professional development (CPD). For determinants with multiple categorical variables (PGT and CPD) where a significant difference was found, post hoc Dunn’s multiple comparison tests were performed to identify the source of differences. All statistical analyses were performed in R (R Version 4.2.3 for Mac, R Core Team 2023).

#### 2.3.2. Use of Culture and Susceptibility by Veterinarians

Respondents were asked whether they had recommended culture and susceptibility testing (C&S) for their described cases, with a binary outcome variable. Some residents also appended written comments regarding the decision not to opt for C&S. With C&S (chosen = 0 or not chosen = 1) as an outcome, variable logistic regression was performed in R using the “glm” function from the base “stats’ package, with antimicrobial treatment chosen for treatment, the predominant field of practice of the respondent, and whether the respondent had PGT or had completed CPD being explored for their association with the outcome. To reduce the effect of infrequently used antibiotics in the models, those used for fewer than eight cases were omitted from the analysis. The null and univariate models were explored, and statistical significance (*p*-values) was determined using the Wald test. Variables with *p* < 0.20 were considered for further consideration in a multivariable model via the backward stepwise elimination of variables with the highest *p*-value > 0.05 and model selection with the smallest Akaike information criterion (AIC). Outcomes of analyses are reported as coefficients, standard error, and odds ratios (ORs) with 95% confidence intervals for the OR of each variable. Variables in the final model were tested for covariance, and diagnostic plots were created using the “glm.diag.plot” function in R to identify unduly influential observations and confirm that standardized residuals were normally distributed (3 of 502 subjects were eliminated from the final model). The goodness of fit of the optimal model was evaluated by calculating the pseudo-r-squared value using the Nagelkerke method [23].

## 3. Results

### 3.1. Survey Respondent Locations and Companion Animal Practice

**Figure 1:**
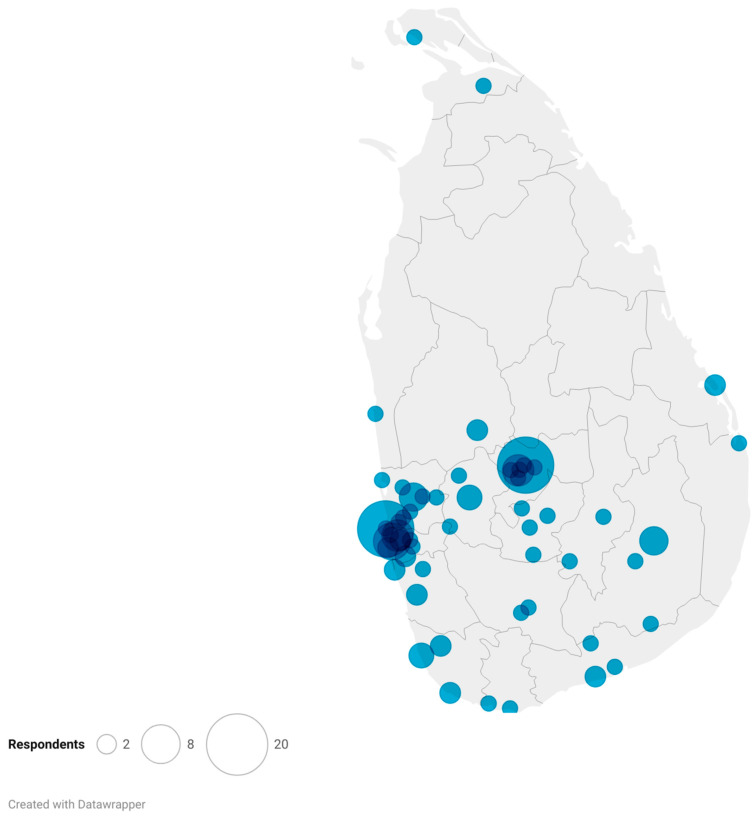
A plot showing the geographic locations within Sri Lanka of 127 Sri Lankan veterinarians who responded to a survey on their use of antimicrobial drugs in companion animals. The large circle to the left encompasses Colombo and the surrounding Western Province, whereas the large circle in the center encompasses Kandy and the Central Province.

There were 134 surveys returned, of which 127 respondents (14.2% of the estimated population) had completed one or more sections of the survey, consistent with a 95% confidence level with a sampling error of 8%. Not all respondents answered all the questions. Therefore, for the results of each question, the denominator reflects the number of veterinarians who responded to that question. Most respondents were from the geographic south of Sri Lanka, with the highest numbers from the population centers of Colombo and the surrounding Western Province, as well as Kandy and the surrounding Central Province (Figure 1). There was limited participation from veterinarians located in the northern provinces, indicating a risk of sampling bias.

Sixty-three percent (80/127) of respondents indicated they were in predominantly companion animal-focused practices (CA), 15.9% (20/127) were in a government practice that predominantly included farm animals (GV), 18.1% (23/127) were in a mixed practice (MP), and 3.1% (4/127) were in other types of practice (e.g., animal charities).

**Figure 2:**
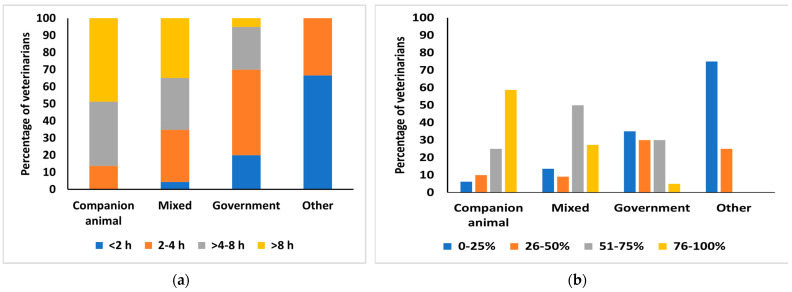
(**a**) The percentage of surveyed veterinarians (126/127) and their hours of daily practice with companion animals by different practice types. (**b**) The percentage of surveyed veterinarians (126/127) and the proportion of their practice associated with companion animals by different practice types.

Companion animal veterinarians more frequently worked with companion animals for more than 8 h daily than veterinarians engaged in mixed, government, or other practice types (Figure 2a), and they were usually working in practices with a predominant companion animal caseload (Figure 2b). Government veterinarians saw a low percentage of companion animal caseloads in their practices.

### 3.2. Survey Respondents’ Education and Continuing Professional Development

**Figure 3:**
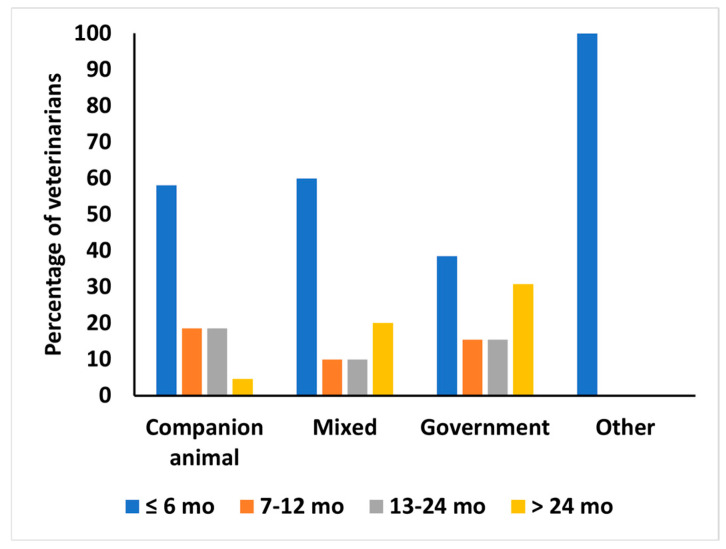
The percentage of surveyed veterinarians (67/127) and the recency of their participation in continuing professional development (months ago) at the time of the survey by different practice types. This included 43/67 companion animal veterinarians, 10 mixed practitioners, 13 government veterinarians, and 1 other practitioner.

All 127 respondents had received their veterinary degree from the University of Peradeniya, Sri Lanka, and 24.4% (31/127) had a postgraduate qualification (PGQ). Those with PGQs included 12.5% (10/70) in CA, 43.5% (10/23) in MP, 50% (10/20) in GV, and 25% (1/4) of veterinarians engaged in other fields of practice. Participation in continuing professional development was indicated by 54.8% (69/126) of respondents, including 65% (13/20), 55% (44/80), 48.8% (11/12), and 33% (1/3) in GV, CA, MP, and other respondents, respectively. One respondent did not answer this question. The recency of participation in continuing professional development (CPD) reported by 67 respondents is shown in Figure 3. Most had completed some form of CPD in the six months before the survey.

### 3.3. Antimicrobial Treatment in Recent Companion Animal Cases

Of the diagnoses specified in the survey, responses to questions on ear infections, urinary tract infections, and acute pyoderma were most frequent (Supplementary Material, Table S2). The median time between examining the patients described and completing the survey ranged from 10 to 30 days (Table S2). Dogs were most frequently described as treated for acute pyoderma, recurrent or deep pyoderma, skin wounds, urinary tract infections, and ear infections. Cats were the species most often treated for abscesses (Table S2).

There were 29 different antimicrobial medications (28 antibacterial and 1 antifungal) used in dogs and cats for different medical conditions, encompassing a range of drug groups (Table 1). Based on the World Health Organization’s criteria [24], 1.7% (13/783) of cases were prescribed drugs authorized for human use only (HUO); 23.9% (187/783) were prescribed highest-priority critically important antimicrobials (HPCIAs); 4.7% (37/783) were prescribed critically important antimicrobials (CIAs); 66.8% (523/783) were prescribed highly important antimicrobials (HIAs); and 1.7% (13/783) were prescribed important antimicrobials (IAs) (Supplementary Table S3). HPCIAs, CIAs, HIAs, and IAs are authorized for humans and animals [18]. Some veterinarians described using multiple drugs in treating the same condition. The medications used for each condition described in the survey are listed in Table 1, along with the frequencies with which respondents used them.

Acute pyoderma was most frequently treated with cefalexin or amoxicillin, recurrent/deep pyoderma was most often treated with cefalexin or amoxicillin/clavulanic acid, and skin wounds were most often treated with cefalexin or amoxicillin. Urinary tract infections were most frequently treated with ciprofloxacin, followed, in descending frequency, by amoxicillin/clavulanic acid and enrofloxacin. Ear infections were most commonly treated with ciprofloxacin, followed, in descending frequency, by cefalexin and enrofloxacin.

### 3.4. Duration of Antimicrobial Treatment

**Figure 4:**
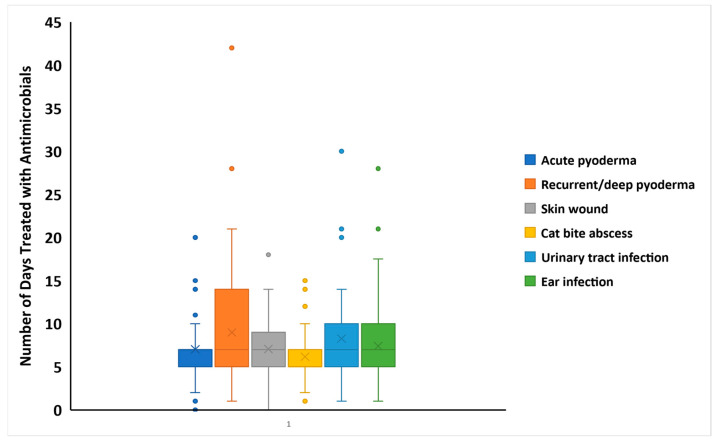
Number of days for which different conditions were treated with antimicrobial drugs by 120/127 surveyed Sri Lankan veterinarians. The line transecting each box is the median, the X within each box is the mean, the upper and lower borders of the box are the third and first quartiles, and the upper and lower whiskers delineate the range of values, exclusive of outliers shown as solid circles.

There was a significant difference in antimicrobial treatment duration among acute pyoderma cases, recurrent or deep pyoderma, canine skin wounds, cat bite abscesses, and urinary tract infections (*p* = 0.0004) (Figure 4). Post hoc analysis found that the duration of antimicrobial treatment for recurrent/deep pyoderma was significantly longer than that used in cases of acute pyoderma (*p* = 0.0013), skin wounds (*p* = 0.0041), or abscesses (*p* = 0.0011). There were no other significant differences.

The duration of antibiotic treatment for all cases prescribed antimicrobials was significantly longer when prescribed by CA respondents than when prescribed by those who identified as MP (*p* < 0.0001) or GV (*p* < 0.0001). There were no significant differences associated with those who had completed PGQs. Those who had indicated the completion of CPD treated animals for longer than those who had not (*p* = 0.039).

### 3.5. Bacterial Culture and Susceptibility Testing

Culture and susceptibility testing was not commonly performed (only 12.4%; 72/579 for all cases). Samples were submitted for testing most often for urinary tract infections (33.7%; 34/101), followed, in descending order, by ear infections (23.1%; 25/108), skin wounds (4.3%; 4/92), abscesses (3.8%; 3/80), acute pyoderma (3.0%; 3/99), and recurrent/deep pyoderma (3.0%; 3/99).

Multivariable analysis identified significant associations between opting for culture and susceptibility testing and the choice of antimicrobial drugs, the medical condition being treated, the practice type, and whether the veterinarian reported completing CPD (Table 2). Some respondents entered free text in which they stated additional barriers to the use of bacterial culture and susceptibility in their practice, including the client’s financial constraints and a lack of access or proximity to facilities for the submission of samples collected from patients for testing.

## 4. Discussion

### 4.1. Survey Respondents

The results of the current study provide useful information on the prescribing practice and use of antibiotics by Sri Lankan veterinarians treating common conditions in companion animals (e.g., cats and dogs). However, the number of participants fell short of the estimated sample size, increasing the sampling error from 5 to 8%. This was due to the distribution of the survey coinciding with the COVID-19 pandemic, which has been identified as a barrier to the One Health surveillance of antimicrobial use and resistance in Sri Lanka [25]. A contemporary online survey focused only on CA veterinarians suffered from similar limits in responses but was unable to estimate the target population before online sampling [19]. Other contemporary South Asian studies on antimicrobial use among veterinarians treating companion animals (or pets) have focused only on CA practitioners, leading to a bias toward urban practitioners [18,19]. In the current study, more than a third of respondents were not solely engaged in companion animal practice, providing antimicrobial use information about a broader cross-section of Sri Lankan practitioners.

The number of Sri Lankan veterinarians engaged in companion animal practice in specific locales is limited, so the collection of demographic information was restricted to geographic location and educational background to protect confidentiality and the respondents’ identities. In agreement with Sandaruwan and Dissanayake (2022), most respondents were located in the most populated provinces [26]. Unsurprisingly, all respondents were educated at Sri Lanka’s only veterinary school, suggesting a pathway for educating emerging graduates on the principles of antimicrobial stewardship. However, postgraduate education was not associated with improved stewardship (e.g., culture and susceptibility testing), likely because there was no evidence that it was focused on antimicrobial stewardship, and few CA practitioners had formal advanced qualifications. Fewer GV than CA and MV practitioners reported participating in CPD in the six months before the survey. However, this difference may reflect the reduced focus on pets in their work and bias associated with the approach to survey distribution at CPD events conducted by the Society of Companion Animal Practitioners. Interestingly, those who had completed CPD were more likely to prescribe a longer duration of antimicrobial therapy, although the reason for this behavior is unclear.

### 4.2. Antimicrobial Treatment in Companion Animal Cases

One concerning finding is the overreliance upon human-use-only microbials and HPCIAs in a quarter of cases. This is concerning when considering the low culture and susceptibility testing submission rate. When this is combined with the use of CIA drugs, the proportion of cases exceeds 30%. In a contemporary survey on Sri Lankan CA veterinarians, more respondents (4% compared to 1.7% in the current study) reported prescribing human-use-only drugs (imipenem and meropenem) [19]. The reported use of HCPIA drugs was also increased (30.1% compared to 23.9% in the current study), and the use of CIA drugs was markedly greater (22.3% compared to 4.7% in the current study) in a study among Indian pet practitioners [18]. Similar challenges have been identified in comparable surveys among CA veterinarians in Portugal [16] and Chile [27], and earlier studies in New Zealand [9] and the United Kingdom [10] report the frequent use of fluoroquinolones and third-generation cephalosporins (HPCIAs) in treatment. The use of these drugs can be effectively reduced through interventions targeted toward improving antimicrobial stewardship [28,29,30,31].

The range of antimicrobials used to treat six commonly encountered conditions was vast. Veterinarians may resort to prescribing antimicrobial agents approved for human use but not animal use if there are insufficient approved options for cats and dogs [32]. Sri Lanka suffers from limited drug availability, demand from clients to prescribe antibiotics, and poor regulation in the pharmaceutical industry, which are significant barriers to implementing antimicrobial stewardship programs [33].

Cases with acute pyodermas, deep or recurrent pyodermas, and skin wounds were most commonly prescribed cefalexin or amoxicillin, with or without clavulanic acid (HIA) [24]. Treatment was prolonged for recurrent/deep pyoderma compared to other conditions studied, indicating the chronicity of many skin conditions. These findings are in agreement with treatment choices for a sample of 54,600 dogs treated for pyoderma by veterinarians in 73 practices across the United Kingdom [34]. Specific case record data were not collected in the current study, so the diagnostic drivers for the treatment are unknown. However, non-responsive infections due to methicillin-resistant *Staphylococcus pseudintermedius* and MDR have become more common, prompting the selection of alternative antimicrobial drugs, as observed in the current study [35]. The validity and effectiveness of the approach taken by Sri Lankan veterinarians in the current study cannot be verified without the routine submission of samples for culture and susceptibility testing [31]. The prescribing patterns observed among veterinarians for these conditions also raise concerns shared by other researchers; when antimicrobial use is not supported by adherence to robust guidelines, antimicrobial resistance and MDR cannot be effectively managed [31,34].

The treatment of abscesses was most often described in cats and similarly treated with amoxicillin, with or without clavulanic acid, or second most commonly, with cefalexin (HIA). Surgical drainage has been shown to shorten antimicrobial therapy with amoxicillin [12], the most common antimicrobial recommended for this condition [27,36], but data on concurrent therapy for abscesses were not obtained. An additional unanswered question concerns the effectiveness of antimicrobial treatment in these cats, as difficulties in administration are well recognized [37,38].

There is concern over the emerging and increasing impact of MDR associated with urinary tract infections in companion animals [39]. Showing the difficulties encountered by Sri Lankan veterinarians in resolving these conditions, they often prescribed human-use-only (5%; imipenem and meropenem) and HPCIA drugs (49%, including ciprofloxacin and enrofloxacin) for cases. The rates of MDR were not determined in the current study, but a recent study on *E.coli* isolates from 21 Sri Lankan dogs with UTIs or endometritis reported a high occurrence (71%; 15/21) of MDR [40]. Although carbapenem resistance in bacterial isolates is generally poorly recognized in animals [41], it has now been reported for *E. coli* isolates from dogs and cats [42,43,44]. Carbapenem resistance is likely to increase if veterinarians continue using this drug class.

Ear infections were treated with HPCIAs and HIAs with a similar frequency (~45%), and < 8% were treated with CIAs. This compares with 27% for HPCIAs in a contemporary Indian study by [18] Otitis externa may result from a primary cause with subsequent infection by commensal bacteria, most often *S. pseudintermedius* and *Malassezia* spp. However, more than 60% of otic *S. pseudintermedius* isolates are reported to be methicillin-resistant, rendering many HIAs ineffective; ~80% of isolates have been identified as MDR [35]. Empirical therapy without susceptibility testing is likely to lead to treatment failures in these cases.

### 4.3. Duration of Antimicrobial Treatment

The recommended treatment duration for superficial pyoderma is two to three weeks, and that for deep pyoderma for a complete cure is four to six weeks [45]. In the current study, the duration of treatment was significantly higher for deep/recurrent pyoderma than for acute pyodermas, skin wounds and abscesses. However, most surveyed veterinarians prescribed antimicrobials for non-deep/recurrent pyodermas for only two weeks and prescribed them for less than one week for acute pyoderma cases. It is unclear whether this is because respondents are unaware of the current guidelines or whether other factors, such as the cost or availability of medication, underpin these findings. For uncomplicated urinary tract infections (UTIs), the duration of treatment recommended by the International Society for Companion Animal Infectious Diseases (ISCAID) is 3–5 days [46]. However, most surveyed veterinarians prescribed antimicrobials for UTIs for more than five days, which is not aligned with ISCAID guidelines. This practice, combined with the low rate of submission for culture and susceptibility testing, may contribute to the development of MDR in UTI-associated bacteria. These concerns are supported by the high occurrence (71%; 15/21) of MDR reported in a recent study on *Escherichia coli* isolates from 21 Sri Lankan dogs with UTIs or endometritis [40]. The findings in the current study related to the duration of antimicrobial treatment emphasize the need for the development, awareness, and application of the prudent use of antimicrobials in the Sri Lankan companion animal practice sector.

### 4.4. Bacterial Culture and Susceptibility Testing

The low percentage of cases in which culture and susceptibility testing was performed (12.4%) is comparable to the 16.7% reported for contemporary human cases in Sri Lanka for which antimicrobials were prescribed [47]. This comparison emphasizes the need for a One Health strategy to address antimicrobial use and resistance in Sri Lanka [25]. Free text contributions to this survey by Sri Lankan veterinarians provide insight into the drivers for low submission rates. The clients’ financial constraints and a lack of rapid antibiogram testing or access or proximity to facilities for the submission of bacterial samples for testing are echoed in the Sri Lankan human health literature [21,25].

The drivers of submission behavior cannot be inferred from retrospective multivariable logistic regression analyses. However, the associations with the antibiotic prescribed, the medical condition being treated, the practice type, and CPD warrant further scrutiny. Tetracyclines were most often used to treat pyoderma. Dermatologic cases encompass a range of causes and disease processes that warrant the performance of pathologic diagnostics, culture and susceptibility testing, and/or molecular techniques before initiating antimicrobial therapy [48]. Culture and susceptibility testing for pyodermas before the prescription of antimicrobials is uncommon, as was the case in the current study [34]. An association with specific types of infections is unsurprising, given the considerations listed above and the recurrent and persistent nature of many urinary tract [46] and ear [49] infections. The role of the practice type requires exploration, but it is possible that GV practitioners are more attuned to recent developments in One Health and AMR surveillance that are driven by government policy [6,21,50]. Education is the cornerstone of the WHO guide to antimicrobial stewardship [45]. The association of CPD with culture and susceptibility testing provides indirect evidence supporting this as a pathway in advancing the principles of antimicrobial stewardship among Sri Lankan veterinarians [19].

### 4.5. Study Limitations

The approach to the survey’s distribution may have resulted in sampling bias. The prolonged distribution period, necessitated by the COVID-19 pandemic, may also have resulted in bias. As this was a study using retrospective case management information, there was a risk of recall bias. Veterinarians in northern geographic locations or rural locations may be underrepresented. Data from many treated cases were incomplete, increasing the risk of error in the multivariable model. The survey did not identify when veterinarians who opted for culture and susceptibility testing performed the testing (i.e., before or after antimicrobial treatment) or whether the test results altered their treatment of cases.

## 5. Conclusions

The results of this cross-sectional survey of Sri Lankan veterinarians show that treating pyodermas, skin wounds, abscesses, urinary tract infections, and ear infections in companion animals is largely empirical, with low sample submission rates for culture and susceptibility as part of clinical decision-making and a limited practice of appropriate antimicrobial stewardship. Guidelines preventing the prescription and supply of human-use-only antimicrobials for cats and dogs are recommended, as well as the further education of veterinarians on the prudent use of antimicrobials. This will support the implementation of a One Heath approach to AMR in Sri Lanka

## Figures and Tables

**Table 1:** List of antimicrobial drugs reported by Sri Lankan veterinarians (n = 120) as being used in treating companion animals for six common medical conditions.

	Medical Conditions Treated with Antimicrobials					
Antimicrobial Drug	Acute Pyoderma n (%)	Recurrent/ Deep Pyoderma n (%)	Skin Wound n (%)	Abscess n (%)	Urinary Tract Infection n (%)	Ear Infection n (%)
Imipenem	1 (0.7)	1 (0.9)	1 (0.9)	1 (1.0)	5 (3.1)	1 (0.7)
Meropenem					3 (1.9)	
Cefixime			1 (0.9)			
Ceftazidime						3 (2.1)
Ceftriaxone				1 (1.0)		
Ciprofloxacin	6 (3.9)	8 (7.1)	3 (2.6)	6 (5.8)	48 (30.0)	37 (26.4)
Enrofloxacin	7 (4.6)	4 (3.6)	4 (3.5)	6 (5.8)	31 (19.4)	22 (15.7)
Amikacin				1 (1.0)	3 (1.9)	1 (0.7)
Clindamycin	1 (0.7)	2 (1.8)				
Framycetin			1 (0.9)			
Gentamicin			1 (0.9)			7 (5.0)
Metronidazole		2 (1.8)	6 (5.2)	6 (5.8)	1 (0.6)	1 (0.7)
Neomycin	2 (1.3)					2 (1.4)
Penicillin/streptomycin				1 (1.0)		
Amoxicillin	38 (24.8)	16 (14.3)	25 (21.7)	35 (34.0)	12 (7.5)	17 (12.1)
Amoxicillin/cefalexin			1 (0.9)			
Amoxicillin/clavulanic acid	19 (12.4)	24 (21.4)	15 (13.0)	19 (18.4)	38 (23.8)	11 (7.9)
Benzylpenicillin		1 (0.9)	1 (0.9)	2 (1.9)		
Cefalexin	54 (35.3)	33 (29.5)	29 (25.2)	14 (13.6)	1 (0.6)	25 (17.9)
Cefoxitin			2 (1.7)	1 (1.0)		
Cefuroxime		2 (1.8)	3 (2.6)	2 (1.9)		
Cloxacillin	9 (5.9)	10 (8.9)	13 (11.3)	4 (3.9)	1 (0.6)	8 (5.7)
Doxycycline	1 (0.7)	1 (0.9)				
Fusidic acid	1 (0.7)					
Penicillin			1 (0.9)			
Tetracycline	5 (3.3)	5 (4.5)	2 (1.7)	1 (1.0)		
Trimethoprim/sulfamethoxazole	5 (3.3)	2 (1.8)	2 (1.7)	1 (1.0)	7 (4.4)	3 (2.1)
Nitrofurantoin	1 (0.7)		1 (0.9)	1 (1.0)	10 (6.3)	
Miconazole ^1^	1 (0.7)	1 (0.9)	1 (0.9)			
None	2 (1.3)		2 (1.7)	1 (1.0)		2 (1.4)
All antimicrobial drugs	153 (100)	112 (100)	115 (100)	103 (100)	160 (100)	140 (100)

^1^ Antifungal agent. Blue, HUO—authorized for human use only; red, HPCIAs—highest-priority critically important antimicrobials; pink, CIAs—critically important antimicrobials; orange, HIAs—highly important antimicrobials; yellow, IAs—important antimicrobials. HPCIAs, CIAs, HOIAs, and IAs are authorized for use in both humans and animals.

**Table 2:** Results of multivariable logistic regression analyses of associations between bacterial culture and susceptibility testing in case management, the most common antimicrobial drugs used, the condition diagnosed, the primary field of practice, and experience of continuing professional development.

Category and Variable	Est. ^1^	SE ^2^	OR ^3^	95% CI ^4^	*p* ^5^ *│>z│*	*p* ^6^
Intercept	−5.9407	1.642	0.0003	0.00005–0.041	0.0003	<0.0001
**Antibiotic**						<0.0001
Aminoglycosides	Ref					
Amoxicillin	−1.708	0.904	0.81	0.03–1.02	0.059	
Amoxicillin–clavulanic acid	0.539	0.753	1.71	0.39–7.79	0.474	
Cefalexin	−1.090	0.872	0.34	0.06–1.84	0.211	
Ciprofloxacin	−0.826	0.742	0.44	0.10–1.92	0.265	
Cloxacillin	0.547	1.107	1.73	0.17–1.45	0.621	
Enrofloxacin	−1.243	0.924	0.29	0.04–1.70	0.178	
Tetracyclines	4.239	1.282	6.93	6.28–1038.60	0.0009	
**Medical condition**						<0.0001
Abscess	Ref					
Acute pyoderma	1.476	1.505	4.38	0.32–160.57	0.327	
Ear infection	4.122	1.398	61.69	6.98–204.67	0.003	
Recurrent/deep pyoderma	0.157	1.534	1.17	0.06–39.26	0.918	
Skin wound	1.598	1.467	4.94	0.40–166.29	0.273	
Urinary tract infection	4.287	1.393	72.71	8.40–2411.34	0.0021	
**Practice type**						<0.0001
Mixed practice	Ref					
Companion animal	0.792	0.465	2.21	0.92–5.87	0.089	
Government	−17.130	1141.9	<0.0001	0–1.93 × 10^12^	0.988	
Other	−16.516	2477.7	<0.0001	0–5.38 × 10^33^	0.995	
**Continuing professional development** (yes/no)	1.380	0.383	3.97	1.91–8.67	0.0003	0.0002

^1^ Coefficient estimate; ^2^ standard error; ^3^ odds ratio; ^4^ 95% confidence interval; ^5^ Wald test *p*-value for category within variable; ^6^ *p*-value for variable in final model; Ref = reference group.

## Data Availability

Ethical restrictions limit the availability of raw data. A deidentified version of the data without geographic information is available upon reasonable request from the corresponding author.

## Supplementary Materials

The following supporting information can be downloaded at: https://www.mdpi.com/article/10.3390/ani15010069/s1, Table S1: Confidential questionnaire regarding the use of antimicrobial drugs in companion animal (pet) practice in Sri Lanka; Table S2: Examination timing relative to survey completion and the duration of the prescribed antimicrobial therapy for dogs and cats treated for common conditions by Sri Lankan veterinarians; Table S3: Antimicrobial medications reported by Sri Lankan veterinarians as being used to treat common conditions in companion animals in a survey on antimicrobial use.
