# Supplementary material for: Antimicrobial Prescribing Practices Among Sri Lankan Veterinarians for Common Medical Conditions in Companion Animals
Source: Animals (Basel). 2024 Dec 31;15(1):69. doi: 10.3390/ani15010069 (PMC11718978; doi:10.3390/ani15010069)
Supplement: Supplementary file 1 [file animals-15-00069-s001.zip › Table S1.pdf]

Table S1. Confidential questionnaire regarding the use of antimicrobial drugs in companion animal (pet) practice in Sri Lanka

---

1. In which city and suburb your practice located?
   ……

2. What is your practice related to?
   - Government veterinarian ☐
   - Companion animal veterinarian ☐
   - Mixed practitioner ☐
   - Other practice ☐

3. Approximately how many hours per day you spent working with companion animals (pets)?
   - None ☐
   - Less than 2 hours ☐
   - 2 to 4 hours ☐
   - >4- 8 hours ☐
   - More than 8 hours ☐

3 What proportion of your time is spent with small animals?
   - 0 - 25% ☐
   - 26 - 50% ☐
   - 51 - 75% ☐
   - 76 - 100% ☐

4. Approximately what is the % of your small animal patients are presented to you as referrals from another veterinarian?

   ………………………………………………….

5. Do you hold any veterinary postgraduate qualifications?

   ………………………………….

6. Level of qualification (PhD. / MVSc./ MSc./ MPhil.)

   ……

7. If yes, please specify your areas.

……
……

8. Did you attend Continuing Professional Development (CPD) conferences or seminars?

……

9. If yes, by whom the CPD programme was organized? (SLVA/ SCAP/ other)

……

10. When did you last attend to a CPD programme related to Companion Animal practice?

- Within last 6 months ☐
- 7-12 months ago ☐
- 13- 24 months ago ☐
- > 24 months ago ☐
- Never ☐

Please provide details about your last case of each of the following infections

Case details	Skin infections				Urinary tract infection	Ear infection
	**Acute or Primary pyoderma**	**Recurrent or Deep pyoderma**	**Dog skin wound**	**Cat bite abscess**		
**Species treated (cat/ dog/ other- please state)**						
When was the case presented (e.g. 2 weeks ago; 1 month ago...)						
Was a bacterial culture and sensitivity test undertaken for this case?						
Did the case resolve after antimicrobial treatment? (Yes/ No/ other- explain and add comments if necessary)						
When did you last see this case (If known)						
**PLEASE PROVIDE THE NUMBER OF DAYS FOR WHICH ANTIMICROBIAL DRUGS WERE ADMINISTERED OR DISPENSED PLEASE PROVIDE THE NUMBER OF DAYS FOR WHICH ANTIMICROBIAL DRUGS WERE ADMINISTERED OR DISPENSED**						
Amikacin						
Amoxicillin						
Amoxiillin-Clavulanic acid						
Cefalexin						
Ciprofloxacin,						
Enrofloxacin						
Gentamicin,						
Imipenem,						
Tetracycline,						
Trimethoprim-sulfamethoxazole						
Nitrofurantoin,						
Cloxacillin,						
Cefoxatin						
Other (Please specify)						
None						

For how long would you routinely treat the following diseases? (More common length of time in days)

Days of treatment	Skin infections				Urinary tract infection	Ear infection
	Primary pyoderma	Recurrent pyoderma	Deep pyoderma	Cat bite abscess		
< 3 days						
3-7 days						
8-14 days						
15-21 days						
>21 days (State the number of days)						

Skin infections

How many cases of skin infections do you **see** in an average working week?

- <1 ....
- 1-5 ....
- 6-10.................
- 11-20................
- 21-40................
- 41-60……………..
- >60……………….

How many cases of skin infections do you **treat with antimicrobials** during an average working week?

- <1 ....
- 1-5 ....
- 6-10.................
- 11-20................
- 21-40................
- 41-60……………..
- >60……………….
- All ………………….

When treating skin infections, when do you recommend bacterial culture and sensitivity testing? (Please tick one or more boxes where it is applicable)

1. In all cases of acute or superficial pypderma ☐
2. In all cases of recurrent or deep pyoderma ☐
3. In all cases of skin wounds ☐
4. Based on the cytology results if a mixed infection is suspected ☐
5. Never ☐
6. Other (Please state) ....

Urinary tract infections

How many cases of urinary tract infections do you **see** during an average working week?

- <1 ....
- 1-5 ....
- 6-10.................
- 11-20................
- 21-40................
- 41-60…………….
- >60……………….

How many cases of urinary tract infections do you **treat with antimicrobials** during an average working week?

- <1 ....
- 1-5 ....
- 6-10.................
- 11-20................
- 21-40................
- 41-60……………..
- >60…………………
- All ………………….

In what circumstances do you recommend urine culture and sensitivities? (Please tick one or more boxes where it is applicable)

1. Uncomplicated urinary tract infections ☐
2. Relapsing or persistent urinary tract infections ☐
3. Never ☐
4. Other (Please state) ☐
   ....

Ear infections

How many cases of ear infections (Otitis externa) do you treat **see** during an average working week?

- <1 ....
- 1-5 ....
- 6-10.................
- 11-20................
- 21-40................
- >40…………………

How many cases of ear infections (Otitis externa) do you **treat with antimicrobials** during an average working week?

- <1 ....
- 1-5 ....
- 6-10.................
- 11-20................
- 21-40................
- >40…………………
- All……………………

For what percentage of ear infections would you recommend bacterial culture and sensitivity testing?

- Never ☐
- < 5% ☐
- 1-5 ☐
- 6-10 ☐
- 11-20 ☐
- >20 ☐
- All ☐

When treating ear infections, when do you recommend bacterial culture and sensitivities? (Please tick one or more boxes where it is applicable)

1. When Gram negative rods are seen in cytology ☐
2. When Gram positive cocci are seen in cytology ☐
3. When yeast organisms are seen in cytology ☐
4. With recurrent ear infections ☐
5. Never ☐
6. Other (Please state)
   ....

☼ ☼ ☼ ☼ ☼ ☼ ☼

Thank you for your cooperation!!
