# Supplementary material for: Antimicrobial Prescribing Practices Among Sri Lankan Veterinarians for Common Medical Conditions in Companion Animals
Source: Animals (Basel). 2024 Dec 31;15(1):69. doi: 10.3390/ani15010069 (PMC11718978; doi:10.3390/ani15010069)
Supplement: Supplementary file 1 [file animals-15-00069-s001.zip › Table S2.pdf]

Table S2. Examination timing relative to survey completion and duration of prescribed antimicrobial therapy for dogs and cats treated for common conditions by Sri Lankan veterinarians (n=120).

Condition diagnosed	Number of animals n (%)					
	Dogs	Cats	NR	Median (days)	IQR (days)	Range (days)
**Acute pyoderma**	47(45.6)	1(1.0)	55(53.4)			
When examined? (n=78)				14	23	0-365
When last rechecked? (n=28)				10	12	0-76
Duration of antimicrobial therapy (n=86)				7	2	0-14
**Recurrent/deep pyoderma**	71(68.9)	2(1.9)	30(29.1)			
When examined? (n=69)				30	61	1-365
When last rechecked? (n=34)				16	54	0-364
Duration of antimicrobial therapy (n=71)				7	9	1-42
**Skin wound**	75(72.8)	1(1.0)	27(26.2)			
When examined? (n=70)				7	23	1-365
When last rechecked? (n=26)				7	18	0-185
Duration of antimicrobial therapy (n=68)				7	5	0-18
**Abscess**	10(9.7)	61(59.2)	32(31.1)			
When examined? (n=65)				14	23	0-180
When last rechecked? (n=23)				13	18	5-198
Duration of antimicrobial therapy (n=62)				7	2	1-15
**Urinary tract infection**	79(76.7)	11(10.7)	13(12.6)			
When examined? (n=83)				14	23	0-365
When last rechecked? (n=33)				13	25	0-159
Duration of antimicrobial therapy (n=87)				7	2	1-30
**Ear infection**	93(90.3)	4(3.9)	6(5.8)			
When examined? (n=90)				10	13	0-180
When last rechecked? (n=32)				7	5	1-45
Duration of antimicrobial therapy (n=85)				7	5	1-28

Numbers parentheses indicate the number of respondents that gave responses to that question. Not all veterinarians responded to questions on all diseases.
