# Supplementary material for: Antimicrobial Prescribing Practices Among Sri Lankan Veterinarians for Common Medical Conditions in Companion Animals
Source: Animals (Basel). 2024 Dec 31;15(1):69. doi: 10.3390/ani15010069 (PMC11718978; doi:10.3390/ani15010069)
Supplement: Supplementary file 1 [file animals-15-00069-s001.zip › Table S3.pdf]

Table S3. Antimicrobial medications reported by Sri Lankan veterinarians as being used to treat common conditions of companion animals in a survey of antimicrobial use.

Importance	Antimicrobial drug	n (%)	Drug group	Comments
HUO	Imipenem	10(1.3)	ß-lactam	Carbapenem
HUO	Meropenem	3(0.4)	ß-lactam	Carbapenem
HPCIA	Cefixime	1(0.1)	ß-lactam	Third generation cephalosporin
HPCIA	Ceftazidime	3(0.4)	ß-lactam	Fourth generation cephalosporin
HPCIA	Ceftriaxone	1(0.1)	ß-lactam	Third generation cephalosporin
HPCIA	Ciprofloxacin	108(13.8)	Fluoroquinolone	
HPCIA	Enrofloxacin	74(9.5)	Fluoroquinolone	
CIA	Amikacin	5(0.6)	Aminoglycoside	
CIA	Clindamycin	3(0.4)	Lincosamide	
CIA	Framycetin	1(0.1)	Aminoglycoside	
CIA	Gentamicin	8(1.0)	Aminoglycoside	
CIA	Metronidazole	16(2.0)	5-nitroimidazole	Antibiotic/antiprotozoal
CIA	Neomycin	4(0.5)	Aminoglycoside	
HIA /CIA	Penicillin/ streptomycin	1(0.1)	ß-lactam /aminoglycoside	ß-lactamase sensitive
HIA	Amoxicillin	143(18.3)	ß-lactam	
HIA	Amoxicillin/cefalexin	1(0.1)	ß-lactam	
HIA	Amoxicillin/clavulanic acid	126(16.1)	ß-lactam	ß-lactamase resistant
HIA	Benzylpenicillin	4(0.5)	ß-lactam	ß-lactamase sensitive
HIA	Cefalexin	156(19.9)	ß-lactam	First generation cephalosporin
HIA	Cefoxitin	3(0.4)	ß-lactam	Second generation cephalosporin
HIA	Cefuroxime	7(0.9)	ß-lactam	Second generation cephalosporin
HIA	Cloxacillin	45(5.8)	ß-lactam	ß-lactamase resistant
HIA	Doxycycline	2(0.3)	Tetracycline	
HIA	Fusidic acid	1(0.1)		Bacteriostatic
HIA	Penicillin	1(0.1)	ß-lactam	ß-lactamase sensitive
HIA	Tetracycline	13(107)	Tetracycline	
HIA	Trimethoprim/ sulfamethoxazole	20(2.6)		Combination
IA	Nitrofurantoin	13(1.7)	Nitrofuran	
N/A	Miconazole	3(0.4)		Antifungal
	None	7(0.9)		
	All antimicrobial drugs	783(100)		

There were 783 medications recommended by 120 Sri Lankan veterinarians for six commonly encountered companion animal medical conditions. HUO —-Authorized for human use only; HPCAI —-Highest priority critically important antimicrobials; CIA —-Critically important antimicrobials.; HIA—-Highly important antimicrobials; IA—-important antimicrobials. HPCIA, CIA, HOIA and IA antimicrobials are authorized for both humans and animals.
